# Spatial Structure and Climatic Adaptation in African Maize Revealed by Surveying SNP Diversity in Relation to Global Breeding and Landrace Panels

**DOI:** 10.1371/journal.pone.0047832

**Published:** 2012-10-16

**Authors:** Ola T. Westengen, Paul R. Berg, Matthew P. Kent, Anne K. Brysting

**Affiliations:** 1 Centre for Development and the Environment (SUM), University of Oslo, Oslo, Norway; 2 Nordic Genetic Resource Centre, Alnarp, Sweden; 3 Department of Biology, Centre for Ecological and Evolutionary Synthesis (CEES), University of Oslo, Oslo, Norway; 4 Department of Animal and Aquacultural Sciences, Centre for Integrative Genetics (CIGENE), Norwegian University of Life Sciences, Aas, Norway; New York State Museum, United States of America

## Abstract

**Background:**

Climate change threatens maize productivity in sub-Saharan Africa. To ensure food security, access to locally adapted genetic resources and varieties is an important adaptation measure. Most of the maize grown in Africa is a genetic mix of varieties introduced at different historic times following the birth of the trans-Atlantic economy, and knowledge about geographic structure and local adaptations is limited.

**Methodology:**

A panel of 48 accessions of maize representing various introduction routes and sources of historic and recent germplasm introductions in Africa was genotyped with the MaizeSNP50 array. Spatial genetic structure and genetic relationships in the African panel were analysed separately and in the context of a panel of 265 inbred lines representing global breeding material (based on 26,900 SNPs) and a panel of 1127 landraces from the Americas (270 SNPs). Environmental association analysis was used to detect SNPs associated with three climatic variables based on the full 43,963 SNP dataset.

**Conclusions:**

The genetic structure is consistent between subsets of the data and the markers are well suited for resolving relationships and admixture among the accessions. The African accessions are structured in three clusters reflecting historical and current patterns of gene flow from the New World and within Africa. The Sahelian cluster reflects original introductions of Meso-American landraces via Europe and a modern introduction of temperate breeding material. The Western cluster reflects introduction of Coastal Brazilian landraces, as well as a Northeast-West spread of maize through Arabic trade routes across the continent. The Eastern cluster most strongly reflects gene flow from modern introduced tropical varieties. Controlling for population history in a linear model, we identify 79 SNPs associated with maximum temperature during the growing season. The associations located in genes of known importance for abiotic stress tolerance are interesting candidates for local adaptations.

## Introduction

According to the Food and Agriculture Organization of the UN, maize (*Zea mays* ssp. *mays*) is the most important food crop in Africa with an annual production of more than 63 million metric tons in 2010 (http://faostat.fao.org/default.aspx). This importance is, however, a relatively recent phenomenon in the history of agriculture. Maize is commonly mentioned as one of the most important crops in the “Columbian exchange” between the New and the Old World in the early 16^th^ century [1]. The intraspecific diversity of maize reflects both the historical introductions of the crop on the continent as well as the local adaptations to a variety of biotic and abiotic conditions. This diversity represents the biological foundation for a substantial part of the food production in Africa and exploring it contributes to research efforts aimed at reducing food insecurity. Unless effective adaptation measures are taken, climate change is predicted to have critical impacts on maize productivity in sub-Saharan Africa [2], [3]. For this reason plant breeding efforts and international development assistance are increasingly focusing on developing and disseminating maize varieties adapted to abiotic stress [4], [5]. These efforts rely on screening of the maize gene pool for traits of relevance to drought tolerance and highlight the important role of *ex-situ* collections as a source of raw material for adaptation. Maize landraces represent a large gene pool for crop improvement [6], but despite the potential for finding adaptations to local agroecological conditions there are few instances where local African varieties have been used in the development of modern varieties for African markets. For African maize diversity to be used in breeding, it will have to be conserved and made available from *ex-situ* collections. While the New World gene pool of maize is well represented in genebanks [7] and well characterized [6], [8] there are few collections of African maize germplasm and limited knowledge about its genetic make-up. Burke et al. [9] reported large geographical gaps in African *ex-situ* collections of maize, in particular when considering the predicted shifts of the climatic zones.

Understanding the spatial structure in African maize diversity is not only interesting from a plant breeding perspective, but also from a socio-economic perspective. Most maize grown by African farmers is still sourced outside the formal seed systems [10] with only 20–30% of the maize grown in sub-Saharan countries grown from certified seeds [11]. The importance of local genetic resources and informal seed systems for adaptation of smallholder agriculture to climate change is starting to receive some attention and research [12]. The vulnerability of agriculture based livelihoods to climate change is directly connected with the accessible genetic resources and characterizing these resources at the seed system level is an important contribution to understand local adaptive capacity.

In this study we use a 50K SNP (single nucleotide polymorphism) array [13] to genotype a selection of maize accessions with a geographically diverse origin, primarily African, with the objective to: 1) trace introductions of maize to Africa from historic and current patterns of gene flow; and 2) scan for signs of adaptations to local climatic conditions during the growing season. The accessions genotyped represent introductions of maize at different temporal scales, including various routes and sources of historic and recent germplasm introductions. In order to make inferences on the origins of the accessions genotyped, we analyzed the selection in the context of two large reference panels representing the global diversity of maize genetic resources; the maize association population [14] and the New World landrace panel [8]. Both panels are major reference populations in the maize community and are uniquely suited for contextualisation of African maize diversity. We compare our SNP results with those obtained with other markers in previous studies of the reference panels and we test if the genetic structure is robust when changing the number of markers. In order to scan for associations between SNP loci and climatic parameters, we perform environmental association analyses [15], [16] applying the general and mixed linear models (GLM, MLM), using climate parameters as dependent variables and controlling for population structure. We discuss the relevance of our results for further exploration and utilization of local genetic resources of maize in Africa.

## Materials and Methods

### Plant material

We analyzed 48 maize accessions obtained from international and national genebanks as well as collected in the field (Table S1). The largest numbers of accessions are from Sudan and Tanzania, two countries selected for their different agro-ecological conditions and their different histories of agricultural development. The remaining accessions are from other African countries except for one European population descending from an early introduction of maize in Spain [17]. Among the local varieties are three accessions of modern varieties grown by some farmers in the sample areas and therefore likely sources of recent gene flow (the open pollinated varieties (OPVs) Staha and TMV1 from Tanzania and the hybrid variety Longe 5 from Uganda). For ease of reference we refer to our sample as the African panel. The seed samples provided by genebanks were randomly drawn from accessions representing the original populations. The seeds sampled in the field were randomly drawn from different ears and plants in farmers' fields or storage. We germinated all samples in growth chambers at the University of Oslo with conditions of 24°C and 12 hours daylight. We harvested leaves of 7-14 days old plants and dried the samples on silica gel. One plant was randomly selected to represent each population according to the procedure in previous continent-wide studies of genetic structure in maize [8], [18], [19]. DNA was extracted from 15 mg leaf samples using E.Z.N.A. plant DNA Mini Kit (Omega Bio-tek, Norcross, GA), according to the manufacturer's protocol. DNA quantity was checked using PicoGreen (Molecular Probes, Eugene, OR), and quality (proportion of high-molecule weight DNA) was assessed by agarose gel electrophoresis.

All necessary permits were obtained for the described field studies. In South Sudan we obtained research and export permit from the Ministry of Agriculture and Forestry. In Tanzania we obtained clearance permit to conduct research through Sokoine University of Agriculture and export of seed samples was permitted by the Ministry of Agriculture, Food and Cooperatives. Sampling in farmers' fields was done under prior informed consent with village authorities and the farmers themselves. All material included in the study was transferred under the terms of the International Treaty on Plant Genetic Resources (http://www.planttreaty.org/), and standard material transfer agreements (http://www.planttreaty.org/content/what-smta) were signed with seed providing genebanks.

### Genotyping

Genotyping was performed according to the manufacturer's instructions using the MaizeSNP50 array and read on an iScan platform (Illumina Inc, San Diego, CA). The genotype results were produced with GenomeStudio Genotyping Module software (v2010.2, Illumina Inc) using a cluster file (MaizeSNP50_B.egt) available on request from Illumina. The array contains assays to 49,585 high quality markers and its development and description is presented by Ganal et al. [13].

### The reference data

The maize Association Panel (AP) is a set of inbred maize lines representing public sector breeding programs around the world, capturing a large proportion of the diversity available for breeders [14], [20]. The AP is developed at Cornell University as a resource for the maize community and has been used in many association studies [21], [22]. The AP is also the source of the 28 diverse founders of the Nested Association Mapping (NAM) panel [23] used to dissect the genetic basis of complex quantitative traits [24], [25]. The AP is well characterized by SSR and SNP markers in previous studies. Based on the Bayesian model-based cluster analysis implemented in STRUCTURE [26], a consistent pattern of three subpopulations and a fourth mixed group has been identified [20], [27]. The three clusters are referred to as non-stiff stalk (NSS), stiff-stalk (SS) and tropical and subtropical (TS) subpopulations. In addition to these groups the popcorn and sweetcorn populations are identified based on knowledge of their distinct phenotype. The MaizeSNP50 array data on the AP was provided by the Panzea (http://www.panzea.org/) team and is available from Cook et al. [25].

The Landrace Panel (LP), used as reference material in this study, is the panel studied by van Heerwaarden et al. [19]. It consists of 1127 landraces plus 100 accessions of the wild ancestor of the domesticated maize, *Zea mays* ssp. *parviglumis,* and 96 accessions of *Zea mays* ssp. *mexicana.* The landrace portion of this panel was used by Vigouroux et al. [8] in their assessment of population structure in New World maize landraces. These populations were selected to cover the pre-Columbian range of maize and represent almost all of the 350 described races in the New World. Van Heerwaarden et al. [19] used Principal Component Analysis (PCA) to cluster the LP into 10 geographically distinct landrace groups using 964 SNPs. With courtesy of the authors we downloaded the LP dataset used in van Heerwaarden et al. [19] from http://www.rilab.org/labstuff/labstuff.html.

### Data analysis

In order to make inferences of the historical and contemporary processes that have shaped the current patterns of genetic diversity, we performed different analyses to identify genetic similarity and variability between populations. We analysed the African panel alone and in combination with the AP and the LP data. Furthermore, in order to test if the structure results are robust when changing the number of markers, we analysed two subsets of the 26,900 PZE-prefix SNPs in the African panel dataset: a selection of every 10^th^ marker (2,691 SNPs) and a selection of every 100^th^ marker (270 SNPs) (hereafter referred to as the 10% and 1% datasets). We matched and merged our data with the reference datasets according to common markers using awk scripts. Since the LP data was generated on a SEQUENOM platform, we extracted our dataset in “top” format from GenomeStudio.

We used the Bayesian Markov chain Monte Carlo (MCMC) clustering model implemented in the program STRUCTURE v2.2 [26] to identify population structure. STRUCTURE assumes a model where there are K clusters, characterized by a set of allele frequencies across unlinked loci. We used the correlated allele frequency and admixture model, suitable for detecting subtle population structure in a widespread, outcrossing species [28]. In separate analyses of the African panel and the combined African panel and AP dataset, we performed nine or more independent runs for each value of K up to a minimum of six groups. For the combined African and LP dataset, we did five independent runs for each value of K from 1 to 15. We used a burn-in of 10^5^ MCMC iterations followed by 2×10^5^ iterations for our African datasets and the LP dataset, while we used a minimum of 5×10^4^ and 10^5^ iterations, respectively, for the datasets containing different combinations of the AP. The STRUCTURE analyzes were done on the Bioportal, University of Oslo (www.bioportal.uio.no). The R package and scripts by Ehrich [29] were used to inform selection of probable and biologically meaningful values of K. The similarities of results (Q matrices) from different runs for the same values of K were calculated according to Nordborg et al. [30]. The ad-hoc measure of delta K, the second order rate of change in the log probability of data between successive K values, was calculated according to Evanno et al. [31]. Since the genetic structure is often hierarchical and different numbers of clusters can be adequate, STRUCTURE was run for main groups separately in a nested analysis.

To verify if the SNP based analysis of population structure in the AP was in agreement with results from previous studies, we used the R package to plot the relationship between Q group membership coefficients and the SSR based equivalents in Flint-Garcia et al. [14] and calculated the Pearson's correlation coefficient, as done in Hamblin et al. [27]. Similarly, to check the agreement between the STRUCTURE results from analysis of the 26,900 dataset and those based on the 10% and 1% subsets as well as the non-overlapping LP-marker dataset, we plotted and calculated the correlation of the results.

To assess the differences in Q group memberships from STRUCTURE analyses between the African clusters, we fitted a linear model to logit-transformed Q group membership values in R. We checked the normality assumption with Q-Q plots and the Shapiro-Wilk test.

We constructed dendrograms to visualize the relationship between accessions and clusters identified with STRUCTURE using Nei's distance [32] and the Neighbor-joining (NJ) tree building algorithm [33] implemented in POWERMARKER v2.7 [34]. Three dendrograms were computed; the first was an accession based NJ tree for the African panel, the second was a NJ tree based on STRUCTURE defined clusters previously identified for the AP by Flint-Garcia et al. [14] and in this study for the African panel, and finally a NJ tree of all three datasets combined based on clusters identified in STRUCTURE. We included the teosinte *Zea mays* ssp. *parviglumis* as outgroup in the latter NJ tree. Bootstrap analysis was performed in POWERMARKER and visualized with the CONSENSE program in the PHYLIP package v3.6 [35]. We edited and coloured the trees in FIGTREE v1.3.1 (http://tree.bio.ed.ac.uk/). Gene diversity (expected heterozygosity) [36] and observed heterozygosity were calculated in POWERMARKER for the combined AP, LP and African panel dataset.

We performed environmental association analyses [15], [16] for three climatic variables across the total 43,963 loci; maximum temperature, minimum temperature and precipitation averaged across the months of the growing season. We performed both the general linear model (GLM) and a mixed linear model (MLM) [37] implemented in TASSEL v.4.0 [38]. As in Eckert et al. [15] we treated the environment variable as a phenotype and the statistical model is:
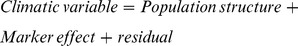



The MLM furthermore includes a pairwise kinship matrix to control for relationship between individuals as a fixed effect. We used the African panel Q matrix based on 26,900 SNPs with the highest likelihood from STRUCTURE analysis at K = 3 to control for population structure in the GLM and included a pairwise kinship matrix (calculated in TASSEL) when fitting the MLM model. Two different datasets were used; one including the 43 African accessions with no known ancestry from modern varieties and one dataset only including 22 accessions with kernel colour differing from white and yellow, using colour as a phenotypic marker for presence of early introduced material. The growing season was determined according to Lobell et al. [2] and the climate variables were obtained with DIVA-GIS v5.2 (http://www.diva-gis.org) using WORLDCLIM 2.5 arc-minutes resolution [39]. In order to adjust the p-values on individual SNPs from the GLM tests, we calculated q-values using the QVALUE R package [40] applying a 1% false discovery rate (FDR) threshold. The location of significantly associated SNPs in relation to known and hypothesized genes in the B73 reference genome was determined with the Panzea marker search database and the MaizeSequence.org (http://www.maizesequence.org) database.

## Results

To obtain a comparable dataset, only SNPs with a call rate >90% were used in the analyses. A total of 43,963 SNPs passed this threshold; 28,477 of these were detected in the public Panzea project (markers with a PZE-prefix). As recommended by Ganal et al. [13] we used only the PZE-prefix SNPs for the diversity analyses as these markers are unbiased for polymorphism between the lines of cultivated maize used for characterization of the MaizeSNP50 array. The final set of SNPs includes the 26,900 PZE-prefix SNPs scored successfully in both the African panel and the Panzea generated AP dataset. The total SNP dataset for the African panel is available in Table S2. The combined African panel and LP dataset contains data on 270 SNPs common to the two datasets while the combined African panel, LP and AP dataset contains 259 common SNPs. All SNP sets used in the analyses are listed in Table S3. The null hypothesis of no geographic structure in the different datasets is firmly rejected. Nested STRUCTURE analyses reveal geographic and historically meaningful patterns of genetic structure both in the African dataset alone and combined with data from the AP and the LP.

### The African panel

**Figure 1:**
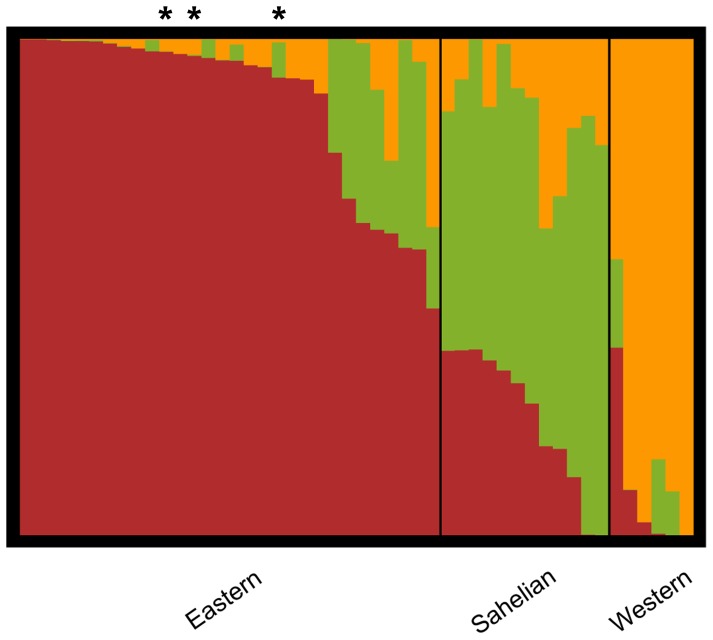
Structure plot of the assignment probabilities in the African panel. Each accession is represented by a bar and the highest Q group membership defines cluster assignment. Asterisks mark the three modern varieties included (from left to right: Staha; TMV1, Longe 5). The plot is based on 26,900 SNPs and the highest probability run for K = 3.

**Figure 2:**
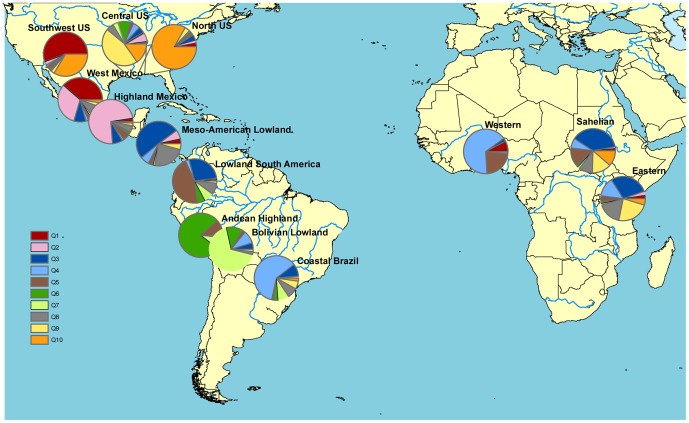
Map of African maize in relation to American landraces. Population structure in the combined African panel and Landrace Panel based on STRUCTURE analysis with K = 10. Each geographic group from van Heerwaarden et al. [19 and each cluster in Africa is represented by a pie diagram whose composition shows admixture coefficients. The position of the pie diagrams on the map are defined by the average latitude and longitude of geographical group or cluster except for the North and Central US group where the pies were moved (indicated by lines) to avoid overlap. Like [19] we excluded the US-derived varieties in South America and we furthermore excluded the Zambian accessions clustering with the Western African cluster and the Spanish accession from the Sahelian cluster.

STRUCTURE analysis of the African panel alone (26,900 SNPs) indicates that the best estimate of K is 3; the likelihood increases up to K = 3 with a decrease in likelihood and an increase in variation and decrease in delta K between runs thereafter (Fig. S1.1, S1.2). A STRUCTURE plot for the 48 accessions is presented in Fig. 1. We name the clusters according to main geographic origin: a Western cluster, including the West African populations plus two Zambian populations; a Sahelian cluster found in Sudan and Chad, including the Spanish population; and an Eastern cluster including all the Tanzanian populations. The names of the clusters and their location on the map (Fig. 2) are based on the average latitude and longitude values of accessions within clusters, excluding the two Zambian accessions from the Western cluster and the Spanish accession from the Sahelian cluster. The K = 3 pattern is consistent between subsets of the data. Comparison of number of assigned accessions (with K = 3) in the 10% and 1% datasets with the full 26,900 African panel dataset is summarized in Table 1 and the full overview of the membership assignments is found in Table S4. Correlations of membership ratios between cluster 1 (Eastern) from the different analyses are strong and significant (Fig. S2a–c). The NJ analysis of the African panel is largely in agreement with the STRUCTURE results, with the accessions belonging to the Western cluster clustering separately from all other accessions and with some intermixture between the accessions belonging to the Sahelian and Eastern clusters (Fig. S3).

**Table 1:** Cluster membership for the African panel.

Assignment level	26,900 PZE-prefix SNPs	10%	1%	270 LP SNPs
>80%	28	28	20	17
>60%	37	33	29*	29*

* in addition to the number of assignments in agreement with the 26,900 PZE-prefix markers, two and one accessions were asigned to a different cluster in the 1% and 270 LP SNP datasets, respectively.

Comparison of number of accessions assigned to three clusters defined by STRUCTURE for the African panel based on three PZE-prefix SNP datasets (26,900 SNPs and subsets of 10% (2691) and 1% (270)), and one 270 non-PZE-prefix SNP dataset (the Landrace Panel SNPs). Assignment level refers to the Q group membership threshold for assignment of an accession to a cluster.

### Relationship with the Association Panel

**Figure 3:**
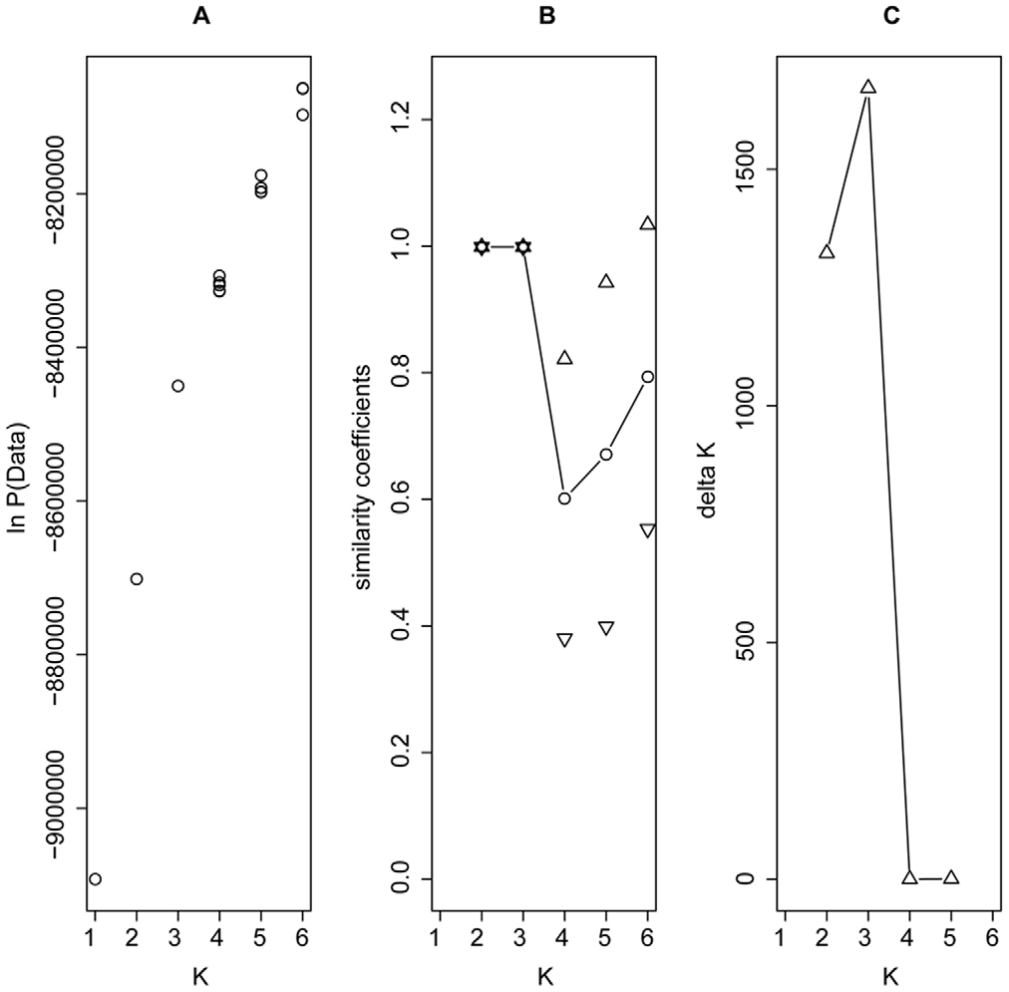
Structure results for the combined African panel and association panel. Plots of STRUCTURE results for the combined African panel and Association Panel showing: a) the Ln (probability of the data) for the values of K from 1 to 6; b) the similarity coefficient for nine different runs per K; and c) the delta K value.

STRUCTURE analysis of the combined African panel and AP dataset (26,900 SNPs) confirms earlier findings about the structure of the breeding material and firmly clusters the African panel within the tropical and subtropical group. The likelihood value increases continuously with no obvious inflection point (Fig. 3a). The similarity coefficient based on comparison of Q matrices from different runs with the same value of K [28] is close to 1 (>0.99) for K values 2 and 3 while it drops significantly for K = 4 and the variation between runs remains high also for higher values of K (Fig. 3b). The highest delta K value (according to [31]) is observed for K value 3 (Fig. 3c). The three clusters identified correspond well with those identified in earlier assessments of the AP; the non-stiff stalk (NSS), stiff-stalk (SS) and tropical and subtropical (TS) subpopulations (Table S5). By plotting the relationship of NSS and TS Q group membership from Flint-Garcia et al. [14] vs. the corresponding Q group memberships from our analysis, we obtain strong correlations (Pearson's R^2^ = 0.94 and 0.96 (p<0.01); Fig. S2d,e), slightly higher than those of Hamblin et al. [27] for similar plots of Q memberships from SNP vs. SSR data.

In the NJ analysis combining the previously identified clusters for the AP [14] and the clusters identified for the African panel, we find very high bootstrap support (100%) for a relationship distinguishing between two main sister groups: the African clusters and the TS cluster from the AP versus the NSS, SS, Popcorn and Sweetcorn clusters. Within the first group the Sahelian and the Western clusters form one group while the Eastern cluster forms a group with the TS cluster (Fig. S4). Considering the STRUCTURE analysis of the African panel in the AP context, we see a strong dominance of the TS defining Q group in all three African clusters (Fig. S5). The NSS and SS defining Q groups are present in all African clusters, but the NSS Q group membership is significantly higher in the Sahelian cluster than in the Western cluster (p<0,001), while the Eastern cluster is intermediary and not significantly different from the two others with respect to the NSS Q group membership.

### Relationship with the Landrace Panel

We matched the data from van Heerwaarden et al. [19] with the data generated in this study in order to investigate the relationship between New World landraces and the African local varieties. We identified 270 common SNPs between the two datasets; none of these are among the 26,900 PZE-prefix SNPs used in the analysis presented above, but several correlation tests confirm that the analysis of the two datasets (26,900 and 270) are in agreement. The correlation between Q1 membership from the full PZE-prefix SNP dataset and the smaller LP-marker dataset in the African panel is strong (R^2^ = 0.87, p<0.01) (Fig. S2a). In the STRUCTURE analysis of the combined African panel and LP, the likelihood shows a steady increase with no clear inflection point (Fig. S1.3). Like in Vigouroux et al. [8] maximal delta K occurred at K = 2, dividing the panel into an Andean cluster vs. the rest, but the similarity coefficient between runs remain high up to K = 5 for which there is a new peak in delta K. K = 5 distinguishes between five broad geographic clusters: 1) West and Highland Mexico; 2) Meso-American Lowlands and Coastal Brazil; 3) Lowland South America; 4) Andean Highland and Bolivian Lowland and 5) Northern American (Fig. S6a). Comparing the K = 5 clustering from this analysis with the K = 4 clustering estimated in Vigouroux et al. [8], we find that the extra cluster is due to a subdivision of the “tropical lowland” cluster into cluster 2 and 3 above. In order to compare STRUCTURE based clustering directly with the 10 geographic groups in van Heerwaarden et al. [19], we consider how the Q groups given for K = 10 correspond to the groups identified by PCA in that study. We find (despite high admixture in all clusters) that there is dominance of a certain Q group in the different predefined American geographic groups with two exceptions: the Meso-American group contains two different Q groups while the South West US cluster is admixed and dominated by the West Mexico Q group and the North US Q group (Fig. 3; Fig. S6b). Focusing on the African panel at K = 5 shows that it is admixed, but dominated by the Meso-American Lowland and Coastal Brazil Q group, the Lowland South America Q group and the Northern American Q group. Sorting the African samples according to the three clusters identified in the STRUCTURE analysis of the African dataset alone, we see that all clusters have the highest affinity with the broad Meso-American Lowland and Coastal Brazil landrace cluster in the LP. There is, however, a tendency of more Northern American representation in the Sahelian and Eastern Africa clusters compared to Western Africa (p<0,001), and more Lowland South America representation in the Western and Sahelian clusters compared to Eastern Africa (p<0,001) (Fig. S6c). The patterns are even clearer when choosing K = 10 in line with the geographic groups identified in van Heerwaarden et al. [19], revealing that the Coastal Brazilian Q group has a significantly higher value in the Western cluster than in the two other clusters (p<0,002); the Meso-American Q groups are higher in the Sahelian cluster than in the Western cluster (p<0,01); the North US Q group is higher in the Sahelian cluster than in the two other clusters (p<0,05); and the Central US Q group is higher in the Eastern cluster than in the two other clusters (p<0,05) (Fig. 2; Fig. S6d).

### Relationship between all three datasets

**Figure 4:**
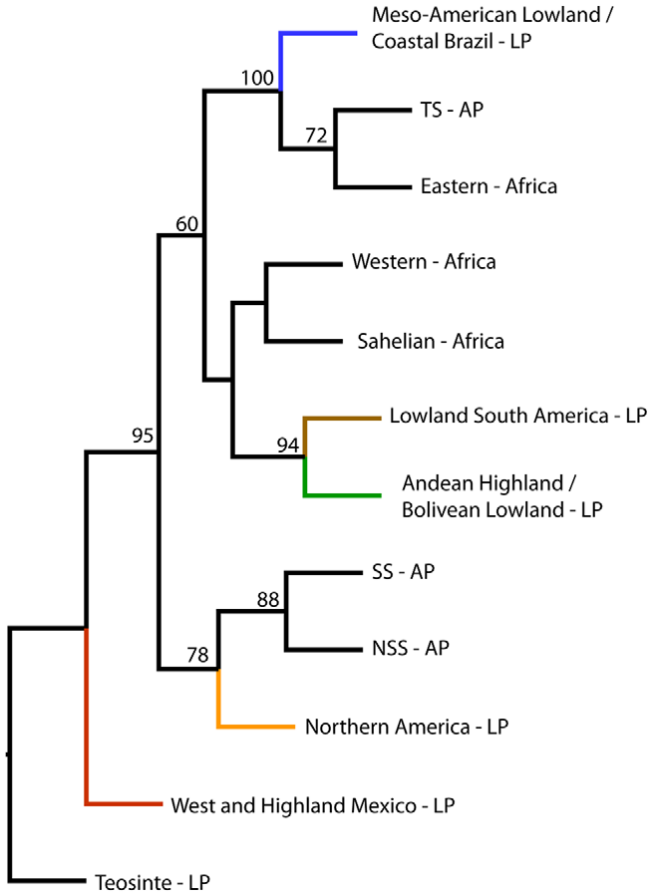
Relationship between African maize and global reference panels. Rooted Neighbor-joining tree of the combined African panel (Eastern, Sahelian, and Western Africa), Association Panel (NSS, SS and TS) and Landrace Panel (branches are named and coloured according to the clusters identified with K = 5 in the STRUCTURE analysis) based on 259 SNPs with bootstrap values in % from 1000 replications.

NJ analysis of the merged dataset (259 SNPs), including the African panel, AP, LP and teosinte as an outgroup, allows further investigation of the relationship between the clusters. The rooted NJ tree in Fig. 4 are based on the clusters defined by STRUCTURE for K = 5 for the LP and K = 3 for the AP and the African panel. The West and Highland Mexico group is sister to the remaining ingroup in 95% of the trees from the bootstrap replicates, reflecting the origin of domesticated maize in this region. The Eastern African samples cluster with the TS cluster from the AP and the Meso-American Lowland and Coastal Brazil cluster from the LP with 100% bootstrap support. The differentiation of the temperate and tropical landraces from the breeding material is less well supported by bootstrap values, but the overall pattern is geographically meaningful and regionally close ecogeographic groups, such as the Lowland South American group and the Andean group, cluster with high bootstrap support. Basic diversity statistics for the panels and clusters are presented in Table 2. At the panel level the African panel and the LP do not display significantly (P< 0.05) different heterozygosity. Heterozygosity is significantly (P<0.05) different between clusters within Africa and between all clusters within the LP, except between the two clusters Lowland South America and Northern America. West and Highland Mexico has significantly (P<0.05) higher heterozygosity than all other LP clusters and only the Eastern African cluster within the African panel has not significantly lower heterozygosity.

**Table 2:** Diversity statistics for panels and clusters.

Panel	Cluster	Genotypes	Gene Diversity	Heterozygosity
AP*		279	0.254 (0.010)	0.00 (0.006)
	TS*	83	0.243 (0.010)	0.002 (0.006)
	NSS*	164	0.246 (0.010)	0.001 (0.006)
	SS*	32	0.150 (0.010)	0.000 (0.006)
LP		1115	0.266 (0.010)	0.183 (0.006)
	West and Highland Mexico	177	0.282 (0.010)	0.222 (0.006)
	Meso-American Lowland/Coastal Brazil	284	0.252 (0.010)	0.198 (0.006)
	Lowland South America	173	0.232 (0.010)	0.184 (0.006)
	Andean Highland/Bolivean Lowland	266	0.186 (0.010)	0.144 (0.006)
	Northern America	215	0.274 (0.010)	0.181 (0.006)
African		48	0.254 (0.010)	0.190 (0.006)
	Eastern	30	0.253 (0.010)	0.218 (0.006)
	Western	6	0.184 (0.010)	0.115 (0.006)
	Sahelian	12	0.236 (0.010)	0.155 (0.006)

Summary statistics, including gene diversity (expected heterozygosity) and observed heterozygosity calculated for the 259 common SNPs in the combined AP, LP and African panel dataset. The standard error of the mean for gene diversity and heterozygosity is given in parenthesis. All clusters within panels are defined by STRUCTURE; K = 3 both for the AP and the African panel and K = 5 for the LP. Statistics were calculated independently on panel- and cluster-level.

* Inbred lines.

### Signs of climatic adaptation

**Figure 5:**
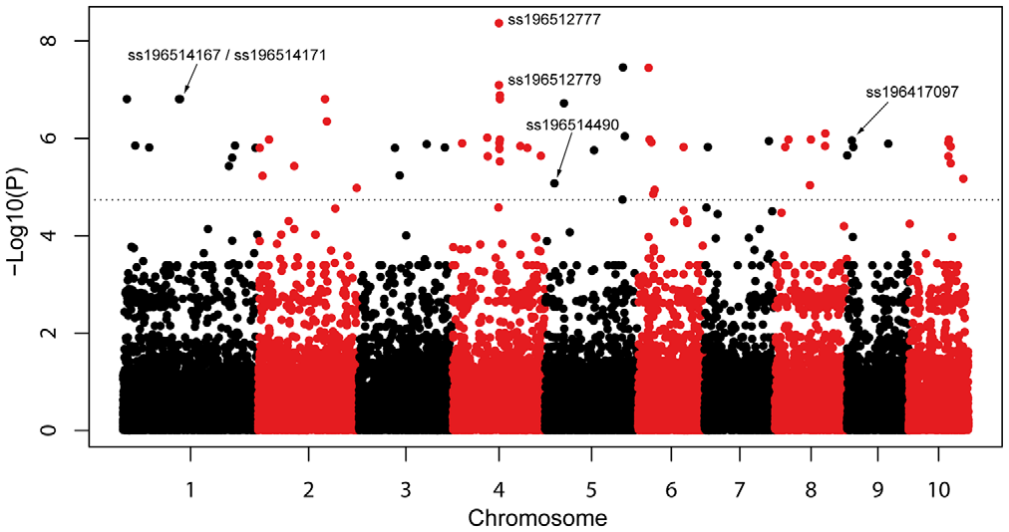
SNP associations with maximum temperature during growing season. Manhattan plot of the log_10_P-values for 43,963 SNPs along the chromosomes (Y-axis) for association with maximum temperature during the growing season. Dotted line indicates significance at 1% false discovery rate (FDR) threshold. Highlighted SNPs are described specifically in the main text.

The environmental association analysis with GLM resulted in identification of loci significantly correlated with climatic variables in the dataset with 22 accessions for which colour is used as a phenotypic marker for presence of early introduced material. None of the models applied reveal significant associations in the dataset with the 43 African accessions in which only accessions with known modern ancestry were excluded. The GLM for associations with maximum temperature during growing season identifies 79 significant SNPs after applying a 1% false discovery rate (FDR) control in the 22 accession dataset (Fig. 5). Likewise, the GLM for association with mean precipitation detects 22 significant SNPs. Analysis with the MLM model does not identify significant associations after FDR control for any of the two datasets analysed. The 79 significant associations from the GLM are listed in Table S6, which also indicates chromosome position and gene affiliation

## Discussion

There is a rich literature on various determinants and aspects of genetic structure in maize. Studies have revealed the domestication history [8], [18], [19], the current population structure of landraces in the centre of origin [41]–[43], the gene flow into landraces from wild relatives [44], from hybrid varieties [45] and from transgenetic varieties [46]. Furthermore, characterization of genetic structure has been used for identification of genetic gaps in breeding material [6], [47], and to control for spurious correlations in association studies [21], [25].

SSRs have traditionally been the marker of choice for diversity studies in maize due to their high information value on an individual marker basis. Since SNPs are biallelic rather than multiallelic they are less informative than SSRs and a relatively higher number of SNPs is needed. Van Inghelandt et al. [48] estimated that for maize the ratio between SNPs and SSRs should be between 7:1 and 11:1 in order to achieve the same level of genetic distance resolution. Accordingly, the 26,900 SNPs used in this study theoretically match the resolution that can be obtained with 2400–3800 SSRs. Only the PZE-prefix markers from the MaizeSNP50 array were used for the phylogeographic analyses, as these markers are not found to be biased towards specific lines of cultivated maize used for characterization of the MaizeSNP50 array [13]. We confirm the agreement of the PZE-prefix SNPs with the predominantly neutral SSRs by finding high correlation between Q group membership in the AP from the two marker systems. The population structure detected with the 26,900 SNPs compared with those detected with SNP subsets down to 270 markers indicates a robust structure with strong genetic differentiation between clusters (Table 1, Table S4, Fig. S2a–c). A slight tendency for the LP-marker dataset to assign plants to the Sahelian cluster identified within the African panel, compared to the PZE-prefix dataset (Table S4), can be due to some ascertainment bias in the non-PZE-prefix markers in this dataset.

### Tracing the population history of maize in Africa

The first introduction of maize to the African continent is undocumented, but even though there are proponents for a pre-Columbian introduction [49], the literature largely focuses on a complex history involving trade routes and cultural diffusion following the Columbian exchange. McCann [50] shows how linguistic evidence is a valuable tool for deconstructing the introduction routes: A typical feature of the traditional African names for maize is their reference to the African relative of maize, sorghum (*Sorghum bicolour*), and indication of where and who it came from; the West African *milho zaburro* means “sorghum of the foreigner” and the Kiswahili term *pemba muhindi* means “Indian sorghum”. Interestingly a widespread name for maize in Nigeria is *masar*, the Arabic name for Egypt, probably obtained from Muslim traders arriving overland from the Arabic north [51], [52]. The Arab introduction of maize was probably an extension of the Columbian exchange. It is documented that Columbus introduced maize from the Caribbean to Spain in 1493, from where it was brought to the Vatican and Italy soon after [53]. Willett [52] describes a plausible route for the red Caribbean flint maize from Seville to Venice and on to Egypt and the Nile Valley from where it spread south and west through present day Sudan and Chad all the way to Nigeria. Thus, according to this theory, West Africa is the meeting point for the two main routes of early entry of maize in Africa; through the Atlantic trade route along the coast and through the inland trade routes with the Arabs. The Western cluster in our study is a group of coloured flint varieties likely to represent early trans-Atlantic introductions and the genetic structure and NJ analysis of this cluster do indeed reflect introduction both from west and east. Considering the structure of the Western cluster in the context of the 10 geographical groups identified in van Heerwaarden et al. [19], we see a strong presence of the Coastal Brazilian Q group (Fig. 2), a pattern in line with Willett's [52] theory about Guianas and Brazil as the source of the first maize introduced by Portuguese and Dutch vessels on the West African coast. According to the STRUCTURE analysis the Meso-American Q groups (K = 10) is more represented in the Sahelian cluster than in the Western cluster. The NJ analysis reveals that within the Sahelian cluster a group of North Sudanese varieties groups with the Spanish traditional variety, supporting the theory about an introduction through mainland Europe in this region (Fig. S3). This North Sudanese group is sister to the branch supporting the Western Africa cluster, with the varieties from Chad in an intermediary position, thus reflecting a northeast-west axis of spread across continental Africa. The rest of the Sudanese varieties are intermixed with the Eastern cluster accessions, a pattern that reflects the meeting of the Arabic and the East African cultures along the border between present day North and South Sudan.

Maize spread rapidly throughout African agriculture and the crop appeared in most areas of the continent within a hundred years after the birth of the Atlantic economy [50]. Under colonial governments varieties developed by professional breeding was introduced for large scale commercial farming, while post-colonial breeding increasingly targeted the African smallholder. In the period from the 1960's to the 1990's many countries experienced a boom in maize production based on genetic improvement supported by national policies and institutions as well as international development assistance [54]. The modern varieties introduced are to a certain degree displacing the local varieties of early introduction ancestry [11], but in many cases the modern and early introduced germplasms are intermixed in the farmers' fields. Informal seed systems play a large role in disseminating recycled improved varieties as well as local varieties with introgression of modern improved germplasm. Farmers' selection and deliberate crossing of improved and local varieties of maize is documented from Mexico [55] and similar practices are shown from Uganda and Tanzania [56]. Since all maize is derived from the same New World gene pool it is not straight forward to distinguish between Q group ancestries from early introductions and introductions of modern varieties, but the combined information from the contextualization of the African panel with both the AP and the LP can resolve some alternative explanations. Breaking the LP comparison ancestry analysis down to the K = 10 level of van Heerwaarden et al. [19] reveals that the Sahelian cluster has more North US ancestry and the Eastern cluster more Central US ancestry than the others. The very low presence of both of the North American LP Q groups in the Spanish accession (Fig. S6c) indicates that the temperate ancestry is not from the early introduction to the Old World, but from more recent introductions. In the case of the Sahelian cluster that scenario is supported by the AP comparison and the significantly higher presence of the NSS ancestry compared to the Western cluster, with the Eastern in an intermediate position (Fig. S5). We suggest that modern introductions of temperate maize as food aid has made a mark on the genetic make-up of maize in the Sahelian cluster and possibly also in the Eastern cluster. The influence of modern vs. early *tropical* ancestry is most difficult to disentangle based on population structure alone; analysed in context of the AP the TS cluster dominates in all African clusters (Fig. S5) and analysed in context of the LP the tropical New World clusters dominate (Fig. S6). However, affinity with modern tropical breeding lines in the Eastern cluster is demonstrated by the >90% assignment of the three OPVs included to this cluster (Fig. 1) and the NJ analysis of all datasets combined suggests that the Eastern cluster is closer to the TS cluster of the AP than the other African clusters (Fig. 4). The grouping of the TS and Eastern cluster with the Meso-American Lowland and Coastal Brazil cluster in the NJ analysis is biologically and historically meaningful since a substantial part of the modern breeding material used, including the OPVs in this study, comes from the International Centre for Maize and Wheat Improvement Centre (CIMMYT) in Mexico and much of this material is derived from tropical landraces from the Meso-American region [6].

Caution must be taken when inferring patterns from the diversity measures presented in Table 2 due to the inherent ascertainment bias. However, the significant decline in heterozygosity from the centre of origin in West and Highland Mexico [18] through Latin America to the Andean Highland is in agreement with earlier assessments of the domestication history [8], [19]. It is notable that the high heterozygosity among West and Highland Mexico landraces is unrivalled in the New World, while it is not significantly higher than the heterozygosity found in the Eastern cluster of the African panel. This probably reflects the geographically and temporally diverse introductions represented in the Eastern cluster.

### Climatic adaptations

Genome-wide SNP data has opened up possibilities for scanning for relationship between genomic sites and climatic variables from the collection sites [15], [16]. While most genetic diversity in the African panel is selectively neutral, reflecting genetic drift and population bottleneck events rather than adaptations, the GLM detected 79 significant SNPs that were associated with maximum temperature during the growing season among the 43,963 SNPs studied in the 22 accessions with colours different from white and yellow (Table S6). The approach of including only accessions whose colour reveal ancestry in early introductions thus proved more successful in identifying potential adaptations than the more inclusive approach where only known modern accessions were excluded. The red, brown, purple and other colours are phenotypic expression of ancestry, either direct or through introgression, in landraces introduced before the introduction of yellow and white improved varieties and it is in the early introduced material that local adaptations have had the most time to occur. The associated SNPs are distributed on all chromosomes (Fig. 5). Among the functional associations detected, four are particularly interesting due to the genes' known role in abiotic stress response: 1) the SNP pair ss196514167/ss196514171 is located in the gene GRMZM2G109814, belonging to the Heat Shock Protein (HSP) 20 gene family whose rice ortholog is known to show increased expression when cells are exposed to elevated temperatures or other stress [57]; 2) the SNP pair ss196512779/ss196512777 is in GRMZM2G107896, encoding argine/serine rich regulators associated with changes in the transcriptome of *Arabidopsis* in response to abiotic stress [58]; 3) the ss196514490 is in the GRMZM2G165901, encoding glycine-rich RNA-binding, abscisic acid (ABA)-inducible protein, which regulates a range of genes with roles in water-stress tolerance in maize [59]; 4) the ss196417097 is in GRMZM2G089713, encoding sucrose synthase, directly affecting floral and seed production under stress conditions [59]. Using GLM, we also detected 22 significant associations with mean precipitation during growing season, while we found no significant associations with minimum temperature. The relatively higher number of associations with maximum temperatures are interesting in light of the finding by Lobell et al. [3], [60] that increase in temperature over a critical level is the most important climate driver of yield loss in crop production in general and maize in particular.

Extending the model to control for a pairwise kinship matrix (K-matrix) in a MLM, the associations were no longer significant after FDR control. The MLM improves statistical power compared to GLM [37] and the lack of significant q-values from the MLM, as well as the low number of accessions included, necessitates a cautious interpretation of the GLM results. Cook et al. [25] similarly did not find significant associations applying a MLM in a genome-wide association study (GWAS) on MaizeSNP50 markers and kernel composition in the AP. Using a candidate gene approach they detected several oil content associations and Cook et al. [25] concluded that true associations with rare alleles in the AP were left undetected in the GWAS due to a lack of statistical power.

The use of climate variables rather than morphological traits in an association analysis was first done in Eckert et al. [15] who identified loci associated with aridity in Loblolly Pine (*Pinus taeda*) applying a linear model and correcting for spurious associations due to confounding of ancestry and aridity using PCA. In a genomic scan of correlations of SNPs with climatic variables Hancock et al. [16] identified a set of candidate climate-adaptive loci in *Arabidopsis thaliana* using a model controlling for population history using a kinship matrix. Hancock et al. [16] proved the environmental association concept by successfully predicting relative fitness among a set of geographically diverse accessions grown in a common environment based on the detected associations. In genetic resource management and use the principle that geographic origin can be considered a proxy for adaptations to eco-geographic variables at the collection site is used in the focused identification of germplasm strategy (FIGS) to select subsets of germplasm from genetic resource collections in order to maximize the likelihood of capturing a specific trait [61]. While FIGS relies on associations between traits and eco-geographic variables, Hancock et al. [16] proved that the principle also works for identification of adaptations at the molecular level. Using statistical methods from association studies we control for shared history and gene flow between the accessions and we suggest that the SNPs situated in genes involved with heat tolerance, carbohydrate production and in the ABA production pathway, detected in the current study to be associated with maximum temperature during growing season, are interesting candidates for local adaptations to climatic stress.

### Relevance for further exploration and utilization of African maize genetic resources

The current study is an example of integration of data from newly genotyped material and publicly available context-data on larger samples. While data from genotyping studies based on SSRs are difficult to analyse outside the particular context of the study, SNP data can be shared meaningfully among labs and studies. We have demonstrated that not only is SNP data generated with the same array technology comparable, but also data generated with different technological platforms. This “technology neutrality” ensures that SNP data does not become redundant as improved technologies become available [62]. This is particularly valuable in a crop species for which thousands of germplasm accessions exist in genebanks with accompanying characterization and evaluation data. As genebanks move towards comprehensive molecular exploration of their *ex-situ* collections [63], the use of stable and reproducible high-throughput technology will add value to all genotyping studies.

Previous studies have reached different conclusions regarding the ability of marker systems to reveal population differentiation based on genetic distance. Warburton et al. [6] found that among the CIMMYT inbred maize lines and OPVs, it was only for closely related lines that SSR variation could resolve relationships. Hamblin et al. [27] found that relationship among the highly diverse inbred AP were difficult to ascertain both with SSRs and SNPs. The pattern is different for landraces. It is the ability of both SSRs and SNPs to detect population differentiation in landraces that allowed Matsuoka et al. [18] to determine the domestication centre and Vigouroux et al. [8] to refine the understanding of the phylogeography in New World maize landraces. The meaningful inference of relationship between the African clusters and the LP clusters, based on genetic distance in 270 markers in this study, indicates that SNPs are well suited to resolve relationships between distantly related local varieties. If the purpose is to use the Q matrix to avoid false positives in association studies, the 26,900 SNPs used here is an unnecessary high number for the task. However, a large number of markers can potentially resolve subtle relationships and disentangle demographic patterns of origin, spread and introgression between highly admixed accessions [19]. Gene flow from multiple introductions have shaped the population structure of African maize and future efforts to resolve demographic patterns should include material with larger geographic coverage, in particular from coastal West Africa and Cape Verde, as well as South Africa and Mozambique.

The high selection pressure from climatic stress in many maize growing regions in Africa is likely to have resulted in local adaptations with potential value for breeding programmes. There are indeed examples of successful use of local African genetic resources in variety development such as the varieties Katumani developed in Kenya, Longe-5 (included in this study) from Uganda and Oba Tanpa from Ghana (K. S. Meseka pers. comm.). Given the urgent need for African maize agriculture to adapt to climate change, innovative and proactive exploration and utilization of climatically adapted local African genetic resources is necessary both in local seed systems and in breeding programmes.

## Supporting Information

Figure S1 **Assessments of Structure results.**
(PDF)

Figure S2 **Plots of the relationship of Q group membership for the same accessions from different datasets.**
(PDF)

Figure S3 **Unrooted accession based NJ tree for the African panel based on 26,900 PZE-prefix SNPs.**
(PDF)

Figure S4 **Unrooted cluster based NJ tree of the combined African and Association Panel**.
(PDF)

Figure S5 **Assignment probabilities for K = 3 in the combined African and Association Panel in predefined African clusters.**
(PDF)

Figure S6 **Estimated population structure in the combined African panel and Landrace Panel.**
(PDF)

Table S1 **List of plant material with source and collection information.**
(PDF)

Table S2 **The total SNP dataset for the 48 accessions in the African panel.**
(TXT)

Table S3 **List of SNPs used in the different analyses.**
(XLSX)

Table S4 **Comparison of Q group membership and cluster assignment between subsets of the African panel.**
(XLS)

Table S5 **Comparison of Q group membership from SSRs and SNPs.**
(XLSX)

Table S6 **SNPs associations with maximum temperature during growing season.**
(PDF)
