# Supplementary material for: Spatial Structure and Climatic Adaptation in African Maize Revealed by Surveying SNP Diversity in Relation to Global Breeding and Landrace Panels
Source: PLoS One. 2012 Oct 16;7(10):e47832. doi: 10.1371/journal.pone.0047832 (PMC3472975; doi:10.1371/journal.pone.0047832)
Supplement: Figure S1 — Assessments of Structure results. (PDF) [file pone.0047832.s001.pdf]

Figure S1

a) b) c)

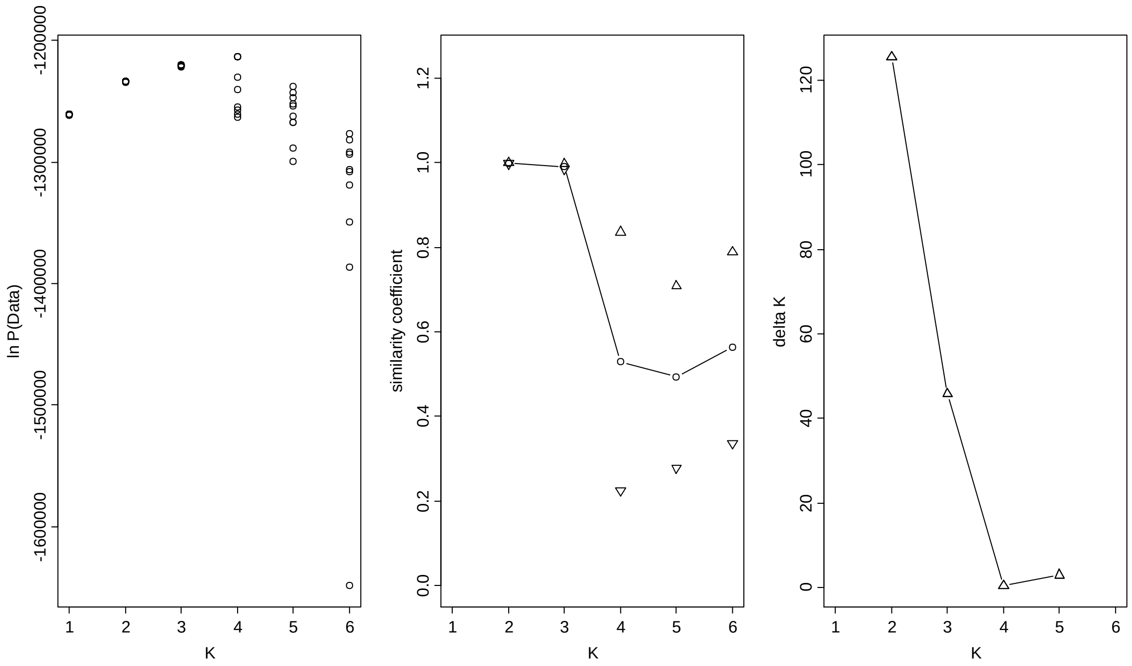

Figure S1.1. *Plots of STRUCTURE results for the African panel (26,900 SNPs) showing: a) the Ln(probability of the data) for the values of K from 1 to 6; b) the similarity coefficient for nine different runs per K, and; c) the deltaK value.*

a) b) c)

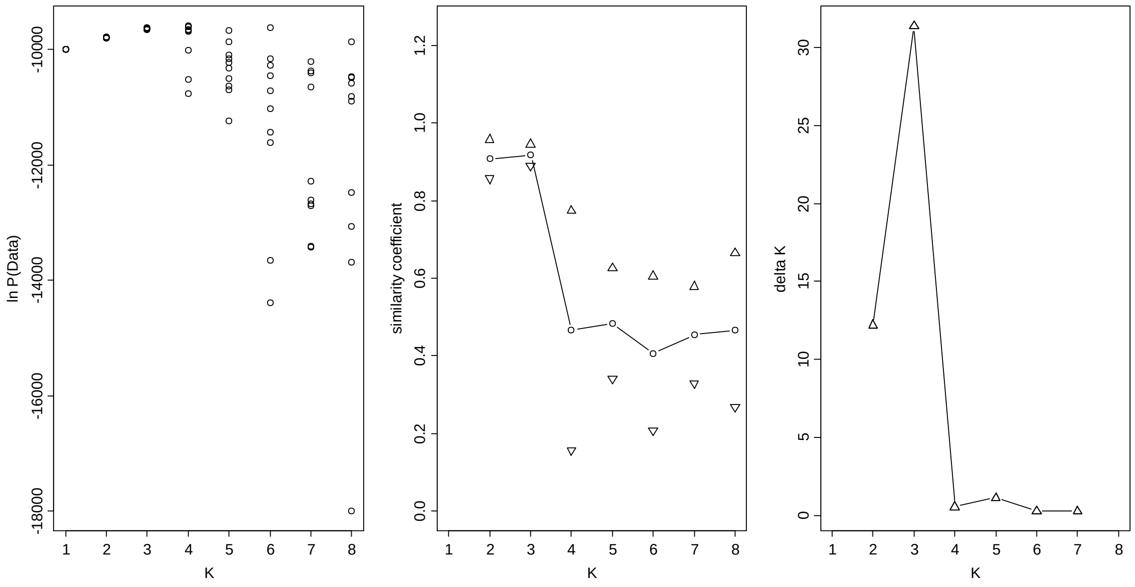

Figure S1.2. Plots of STRUCTURE results for the African panel (270 SNPs common with Landrace Panel) showing: *a) the Ln(probability of the data) for the values of K from 1 to 8; b) the similarity coefficient for ten different runs per K, and; c) the deltaK value.*

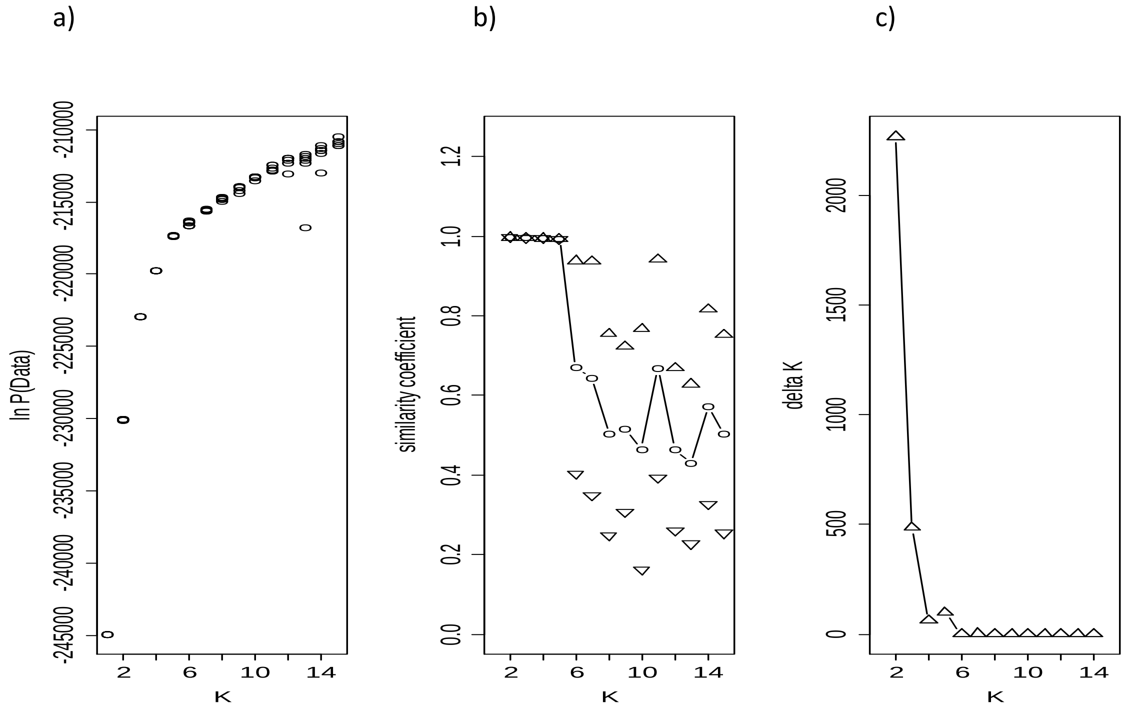

Figure S1.3. *Plots of STRUCTURE results for the for fifteen different values of K from 1 to 15 for the combined African panel and Landrace Panel (270 common SNPs) showing: a) the Ln(probability of the data) for the values of K from 1 to 15; b) the similarity coefficient for five different runs per K, and; c) the deltaK value.*
