# Supplementary material for: Spatial Structure and Climatic Adaptation in African Maize Revealed by Surveying SNP Diversity in Relation to Global Breeding and Landrace Panels
Source: PLoS One. 2012 Oct 16;7(10):e47832. doi: 10.1371/journal.pone.0047832 (PMC3472975; doi:10.1371/journal.pone.0047832)
Supplement: Figure S2 — Plots of the relationship of Q group membership for the same accessions from different datasets. (PDF) [file pone.0047832.s002.pdf]

Figure S2

a)

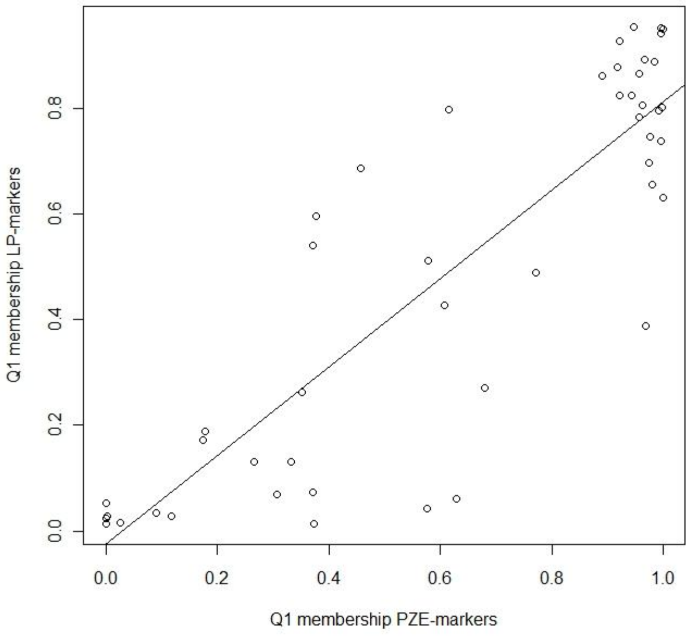

R^2=0.87 (p<0.01)

b)

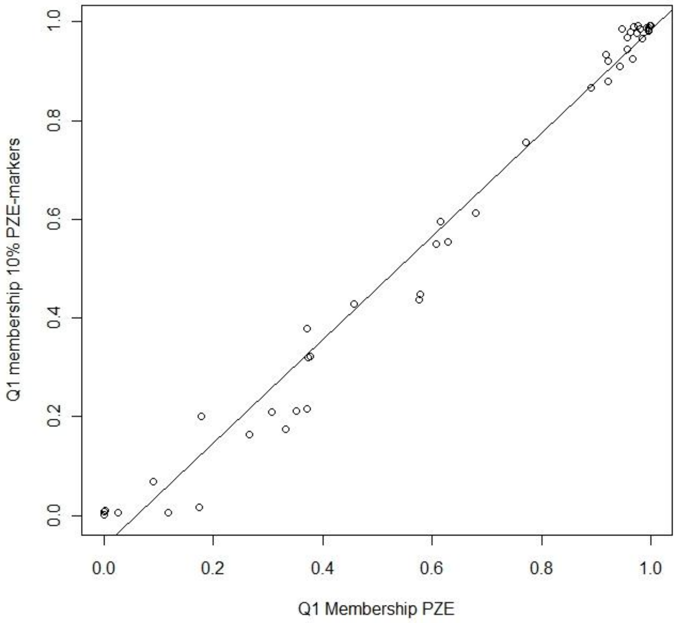

R^2=0.99 (p<0.01)

c)

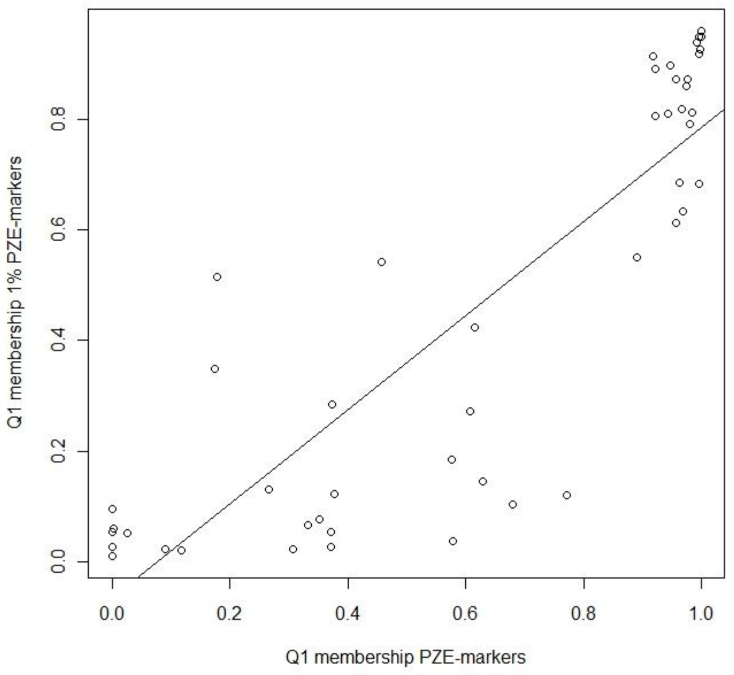

R^2=0.86 (p<0.01)

d)

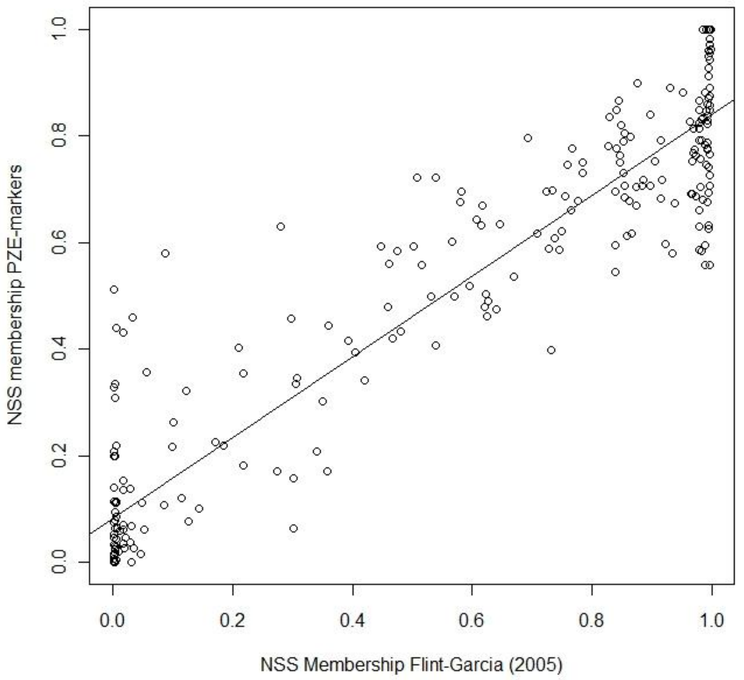

R^2=0.94 (p<0.01)

e)

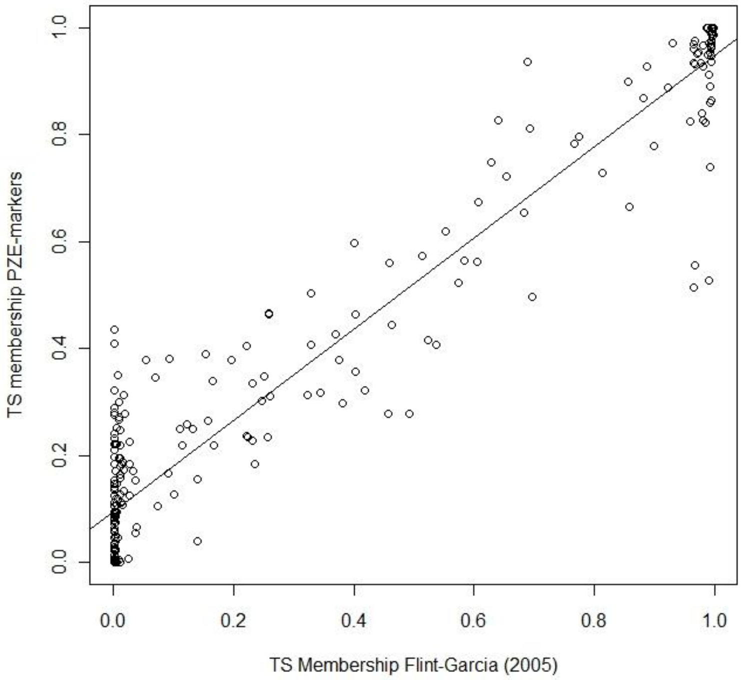

R^2=0.96 (p<0.01)

Fig. S2 Plots of the relationship of Q group membership from different STRUCTURE (Pritchard et al. 2000) analyses: a, b, c) Between Q group membership in cluster 1 (Eastern) in the African panel for the full 26,900 Panzea markers and the Landrace Panel markers, the 10% subset and the 1% subset of the Panzea markers, respectively; d, e) Between accessions in the NSS and TS clusters identified in the Association Panel in Flint-Garcia et al. (2005) vs. the corresponding Q group memberships from our analysis based on 26,900 Panzea markers (PZE-prefix). The correlation coefficient (Pearson's R^2) is given for all plots.
