# Supplementary material for: Spatial Structure and Climatic Adaptation in African Maize Revealed by Surveying SNP Diversity in Relation to Global Breeding and Landrace Panels
Source: PLoS One. 2012 Oct 16;7(10):e47832. doi: 10.1371/journal.pone.0047832 (PMC3472975; doi:10.1371/journal.pone.0047832)
Supplement: Figure S4 — Unrooted cluster based NJ tree of the combined African and Association Panel. (PDF) [file pone.0047832.s004.pdf]

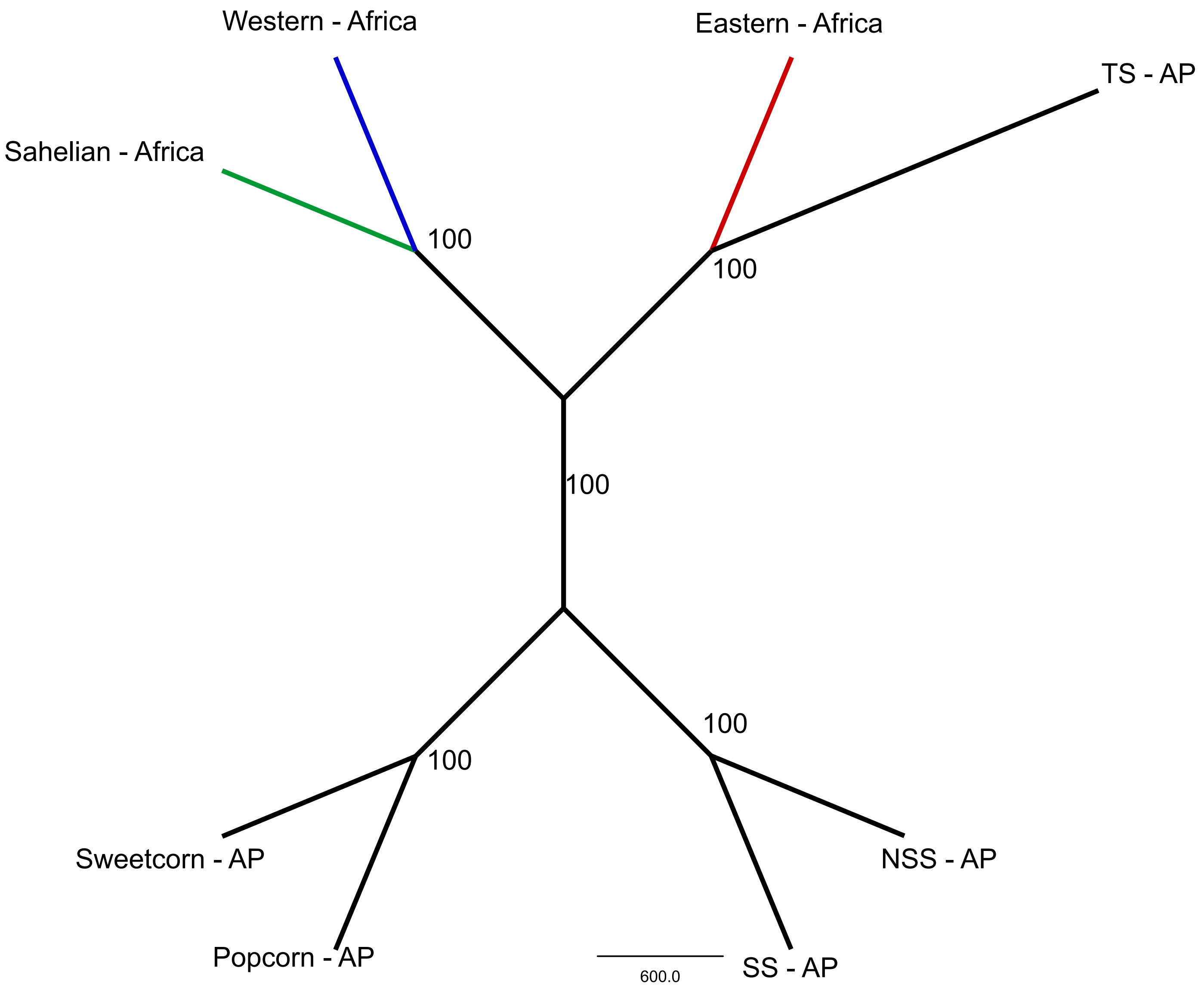

Fig. S4 Unrooted NJ tree of the combined African and Association panel dataset based on 26,900 PZE-prefix SNPs. The tree is based on clusters defined by STRUCTURE analysis of the Association Panel (Flint-Garcia *et al.* 2005) and the African panel (including only accessions with >60% assignment to a Q group and 20 randomly selected lines representing the larger clusters). Bootstrap values in % from 1000 replications.
