# Supplementary material for: Spatial Structure and Climatic Adaptation in African Maize Revealed by Surveying SNP Diversity in Relation to Global Breeding and Landrace Panels
Source: PLoS One. 2012 Oct 16;7(10):e47832. doi: 10.1371/journal.pone.0047832 (PMC3472975; doi:10.1371/journal.pone.0047832)
Supplement: Figure S5 — Assignment probabilities for K = 3 in the combined African and Association Panel in predefined African clusters. (PDF) [file pone.0047832.s005.pdf]

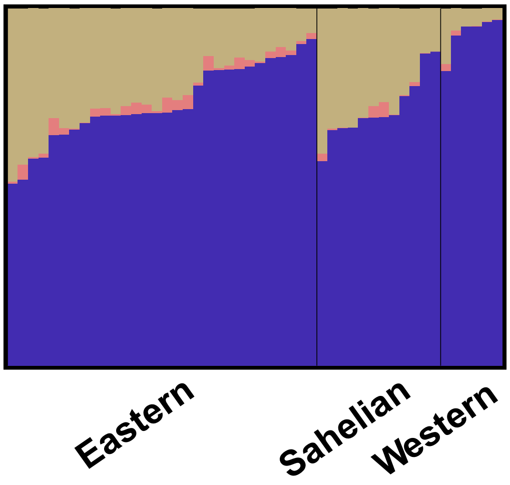

Fig. S5. Assignment probabilities for K=3 (TS (blue), NSS (grey), SS (pink)) in the combined African and Association Panel applied on clusters defined by K=3 in the African Panel alone.
