# Supplementary material for: Spatial Structure and Climatic Adaptation in African Maize Revealed by Surveying SNP Diversity in Relation to Global Breeding and Landrace Panels
Source: PLoS One. 2012 Oct 16;7(10):e47832. doi: 10.1371/journal.pone.0047832 (PMC3472975; doi:10.1371/journal.pone.0047832)
Supplement: Figure S6 — Estimated population structure in the combined African panel and Landrace Panel. (PDF) [file pone.0047832.s006.pdf]

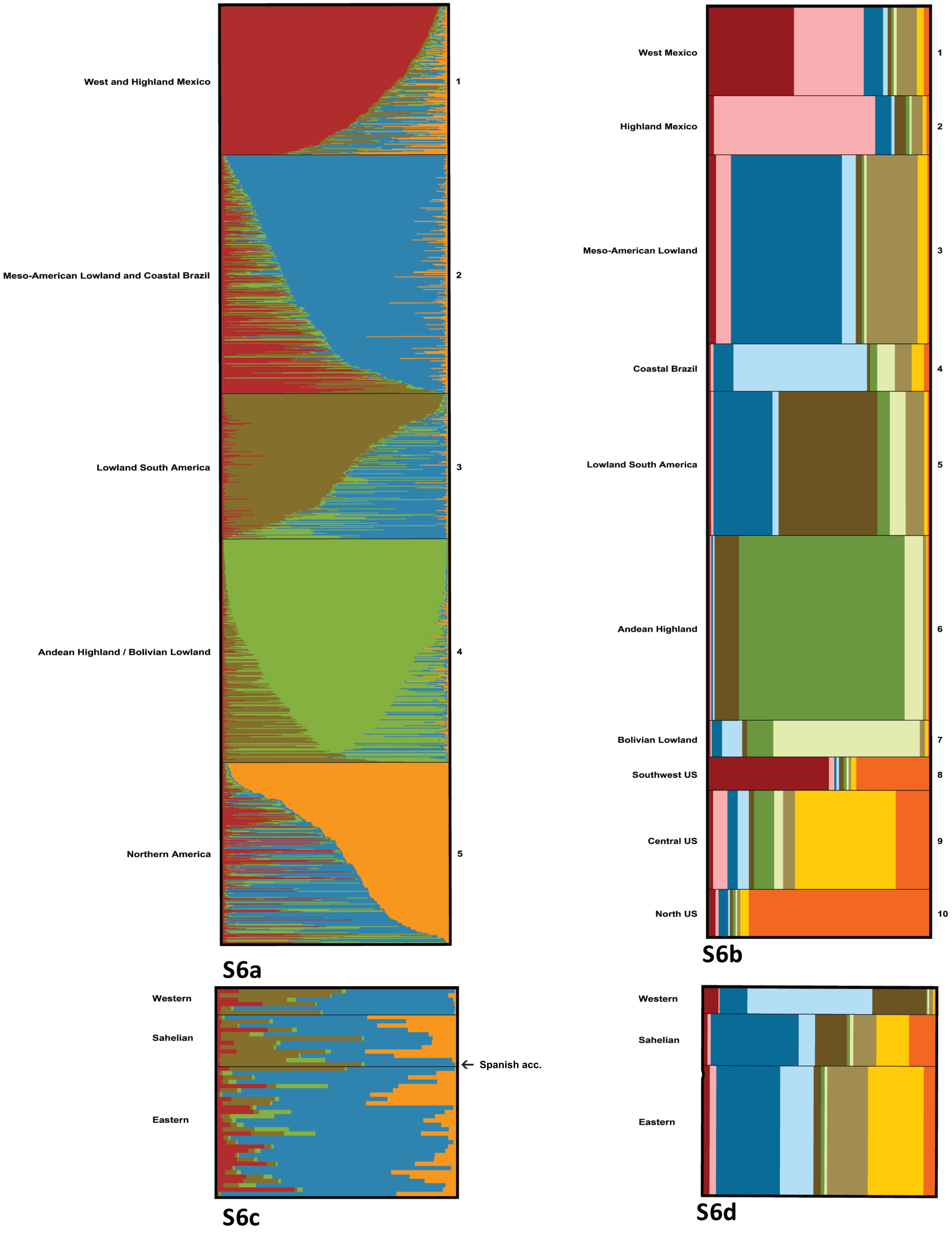

Fig. S6 Population structure in the African panel and Landrace panel (LP) (van Heerwaarden et al. 2011) defined by STRUCTURE. a) Population structure in the combined dataset for K=5 (Each line represents one accession); b) Population structure in the combined dataset for K=10 (The named geographic groups are identified and named by PCA analysis in van Heerwaarden et al. (2011) and the Q groups membership is averaged within geographic group); c) Population structure in the African panel for K=5 (The three named clusters are defined by STRUCTURE analysis of the African samples alone); d) Population structure in the African panel for K=10 applied on the three clusters identified in African panel. Visualization was done by DISTRUCT v1.1 (Rosenberg 2004).
