# Supplementary material for: Spatial Structure and Climatic Adaptation in African Maize Revealed by Surveying SNP Diversity in Relation to Global Breeding and Landrace Panels
Source: PLoS One. 2012 Oct 16;7(10):e47832. doi: 10.1371/journal.pone.0047832 (PMC3472975; doi:10.1371/journal.pone.0047832)
Supplement: Table S1 — List of plant material with source and collection information. (PDF) [file pone.0047832.s007.pdf]

Table S1 List of plant material with source and collection information.

Name in study	Accession Name	Source	Country	Status	Lattitude	Longitude
Ben1	112	IITA	Benin	LV	10.80	3.22
Ben2	118	IITA	Benin	LV	9.72	3.22
Ben3	124	IITA	Benin	LV	8.47	3.22
Gha1	685	IITA	Ghana	LV	10.05	-3.22
Som1	1410	IITA	Somalia	LV	2.47	44.97
Som2	1378	IITA	Somalia	LV	0.08	42.33
Som3	1420	IITA	Somalia	LV	3.78	42.53
Sud1	HSD 3537	ARC Sudan	Sudan	LV	14.00	29.75
Sud2	HSD 3539	ARC Sudan	Sudan	LV	14.00	29.75
Sud3	HSD 3540	ARC Sudan	Sudan	LV	14.00	29.75
Sud4	HSD 4291	ARC Sudan	Sudan	LV	0.51	31.42
Sud7	HSD 4407	ARC Sudan	Sudan	LV	11.85	30.85
Sud8	HSD 4787	ARC Sudan	Sudan	LV	10.88	29.6
Sud9	HSD 4811	ARC Sudan	Sudan	LV	11.23	29.47
Sud10	HSD 5008	ARC Sudan	Sudan	LV	19.17	30.45
Sud11	HSD 5010	ARC Sudan	Sudan	LV	18.03	30.95
Sud12	HSD 5012	ARC Sudan	Sudan	LV	19.25	29.00
Sud13	HSD 5519	ARC Sudan	Sudan	LV	11.45	34.15
Sud14	HSD 5521	ARC Sudan	Sudan	LV	11.28	34.12
Sud15	HSD 5533	ARC Sudan	Sudan	LV	11.53	34.38
Sud16	HSD 7046	ARC Sudan	Sudan	LV	18.42	37.72
Sud17	HSD 10484	ARC Sudan	Sudan	LV	17.38	33.88
Sud18	GPS58	Collected	Sudan	OPV	4.12	32.20
Sud19	GPS62	Collected	Sudan	LV	4.87	32.62
Cha1	321	IITA	Chad	LV	8.97	18.60
Cha2	315	IITA	Chad	LV	8.77	16.25
Cha3	335	IITA	Chad	LV	10.45	16.93
Tan1	TZOW2	Collected	Tanzania	LV	-6.95	37.30
Tan2	TZOW5	Collected	Tanzania	LV	-6.95	37.30
Tan3	TZOW6	Collected	Tanzania	LV	-6.95	37.30
Tan4	TZOW7	Collected	Tanzania	LV	-6.95	37.30
Tan5	TZOW9	Collected	Tanzania	LV	-6.95	37.30
Tan6	TZOW12	Collected	Tanzania	LV	-6.95	37.30
Tan7	TZOW13	Collected	Tanzania	LV	-6.95	37.30
Tan8	TZOW18	Collected	Tanzania	LV	-6.18	36.60
Tan9	TZOW20	Collected	Tanzania	LV	-6.18	36.60
Tan10	TZOW21	Collected	Tanzania	LV	-6.18	36.60
Tan11	TZOW22	Collected	Tanzania	LV	-6.18	36.60
Tan12	TZOW25	Collected	Tanzania	LV	-6.18	36.60
Tan13	TZOW29	Collected	Tanzania	LV	-6.18	36.60
Tan14	TZOW31	Collected	Tanzania	LV	-6.18	36.60
Tan15	TZOW34	Collected	Tanzania	LV	-6.18	36.60
Tan16	TMV1	Collected	Tanzania	OPV	n/a	n/a
Tan17	Staha	Collected	Tanzania	OPV	n/a	n/a
Zam1	1514	IITA	Zambia	LV	-9.75	31.50
Zam2	1516	IITA	Zambia	LV	-9.75	28.83
Zam3	251	IITA	Zambia	LV	-12.50	26.00
Spa1	SP8	CSIC Spain	Spain	LV	n/a	n/a

ARC Sudan, Agricultural Research Corporation Wad Medani
IITA, International Institute of Tropical Agriculture
CSIC Spain, Mision Biologica de Galicia
Collected, collected by OW

LV=Local variety
OPV=Open Pollinated Variety
