# Supplementary material for: Spatial Structure and Climatic Adaptation in African Maize Revealed by Surveying SNP Diversity in Relation to Global Breeding and Landrace Panels
Source: PLoS One. 2012 Oct 16;7(10):e47832. doi: 10.1371/journal.pone.0047832 (PMC3472975; doi:10.1371/journal.pone.0047832)
Supplement: Table S6 — SNPs associations with maximum temperature during growing season. (PDF) [file pone.0047832.s012.pdf]

Table S6. SNPs association with maximum temperature during growing season with genomic location and brief description of transcript. Only significant associations with q < 0,01 controlling for false discovery rate (FDR) are included. *Intergenic SNPs in linkage disequilibrium (r^2>0.10, p< 0.05) with adjacent genic

SNP	Original Name	Chromosome	Position	p-value	Q-value	Gene	Description
ss196422342	PZE-101011230	1	6782009	1.55E-07	0.0003		
ss196422834	PZE-101035008	1	22645687	1.40E-06	0.0011	GRMZM2G055172	Putative uncharacterized protein
ss196423816	PZE-101065758	1	48688790	1.55E-06	0.0011	GRMZM2G013783	AMP-binding proteinPutative uncharacterized protein
ss196514167	SYN26764	1	120247717	1.55E-07	0.0003	GRMZM2G109814	Hsp20/alpha crystallin family protein
ss196514171	SYN26766	1	120248062	1.55E-07	0.0003	GRMZM2G109814	Hsp20/alpha crystallin family protein
ss196522157	SYN36727	1	121487842	1.55E-07	0.0003	GRMZM2G134985	SA: Zea mays contig25349, mRNA sequence
ss196522155	SYN36726	1	121490520	1.55E-07	0.0003	GRMZM2G134985	TSA: Zea mays contig25349, mRNA sequence
ss196426781	PZE-101111506	1	121495993	1.55E-07	0.0003	GRMZM2G134985	TSA: Zea mays contig25349, mRNA sequence
ss196426783	PZE-101111507	1	121496734	1.55E-07	0.0003	GRMZM2G134985	SA: Zea mays contig25349, mRNA sequence
ss196530936	ZM013292-0268	1	121509838	1.55E-07	0.0003	GRMZM2G401848	Peptidyl-prolyl cis-trans isomerase (EC 5.2.1.8)
ss196426797	PZE-101111526	1	121520552	1.55E-07	0.0003		LD*
ss196426799	PZE-101111529	1	121528735	1.55E-07	0.0003		LD*
ss196426811	PZE-101111646	1	121700893	1.55E-07	0.0003		LD*
ss196426815	PZE-101111671	1	121741903	1.55E-07	0.0003		LD*
ss196431500	PZE-101198638	1	246815369	3.69E-06	0.0022	GRMZM2G034503	Putative uncharacterized protein
ss196520180	SYN34253	1	253773132	2.50E-06	0.0016	GRMZM2G342105	TSA: Zea mays contig10757, mRNA sequence
ss196514524	SYN272	1	260146963	1.41E-06	0.0011		
ss196420264	PUT-163a-60399514-2998	1	296875316	1.59E-06	0.0011	GRMZM2G035017	40S rib. prot. S4 Putative uncharacterized protein
ss196419902	PUT-163a-60346998-2564	1	296875324	1.59E-06	0.0011	GRMZM2G035017	40S rib. prot. S4 Putative uncharacterized protein
ss196524529	SYN4145	2	1774988	1.57E-06	0.0011	GRMZM2G044096	Putative uncharacterized protein
ss196433451	PZE-102013856	2	5997113	5.97E-06	0.0034		
ss196527239	SYN7473	2	15972959	1.05E-06	0.0011		
ss196435920	PZE-102083292	2	70334715	3.69E-06	0.0022	GRMZM2G143205	Putative uncharacterized protein
ss196528019	SYN8403	2	163564609	1.55E-07	0.0003	GRMZM2G083972	Putative uncharacterized protein
ss196419559	PUT-163a-4688495-2131	2	169557589	4.49E-07	0.0008	GRMZM2G016066	Photosystem I reaction center subunit IV A
ss196508907	SYN20259	2	233749580	1.04E-05	0.0056	GRMZM2G037614	Putative uncharacterized protein
ss196445369	PZE-103060940	3	108555340	1.56E-06	0.0011		
ss196446596	PZE-103074217	3	122598889	5.79E-06	0.0033		
ss196446598	PZE-103074218	3	122598912	5.79E-06	0.0033		
ss196449272	PZE-103125767	3	183323668	1.31E-06	0.0011		
ss196450477	PZE-103170973	3	218117031	1.54E-06	0.0011	GRMZM2G073460	Clone EL01N0551A02.c mRNA sequence
ss196451835	PZE-104019915	4	20906454	1.26E-06	0.0011		
ss196454783	PZE-104053611	4	85706015	9.73E-07	0.0011	AC182105.3_FGT006	Putative uncharacterized protein
ss196454910	PZE-104055122	4	90626084	2.33E-06	0.0015	GRMZM2G000801	Putative uncharacterized protein
ss196512779	SYN25036	4	135650694	8.10E-08	0.0003	GRMZM2G107896	Splicing factor, arginine/serine-rich 7
ss196512777	SYN25034	4	135650949	4.26E-09	0.0002	GRMZM2G107896	Splicing factor, arginine/serine-rich 7
ss196456387	PZE-104069096	4	137408632	1.64E-06	0.0011		
ss196513411	SYN25809	4	137568454	1.62E-06	0.0011	GRMZM2G041842	TSA: Zea mays contig57940, mRNA sequence
ss196456391	PZE-104069157	4	137569053	1.24E-06	0.0011	GRMZM2G041842	TSA: Zea mays contig57940, mRNA sequence

ss196456395	PZE-104069159	4	137569861	1.55E-07	0.0003	GRMZM2G041842	TSA: Zea mays contig57940, mRNA sequence
ss196456397	PZE-104069164	4	137572872	1.32E-07	0.0003	GRMZM2G041842	TSA: Zea mays contig57940, mRNA sequence
ss196456401	PZE-104069201	4	137608892	1.55E-07	0.0003		LD*
ss196456403	PZE-104069229	4	137658355	1.05E-06	0.0011		LD*
ss196456407	PZE-104069242	4	137788955	2.95E-06	0.0018		LD*
ss196456411	PZE-104069279	4	137929388	1.55E-07	0.0003		LD*
ss196456415	PZE-104069344	4	137981662	1.55E-07	0.0003		
ss196458434	PZE-104112413	4	187595277	1.45E-06	0.0011	GRMZM2G059381	AMP-binding protein
ss196459145	PZE-104126470	4	204416923	1.59E-06	0.0011		
ss196460378	PZE-104148574	4	235704291	2.28E-06	0.0015		
ss196514490	SYN27136	5	14072511	8.34E-06	0.0046	GRMZM2G165901	Glycine-rich RNA-binding, abscisic acid-inducible protein
ss196517393	SYN30636	5	35008584	1.91E-07	0.0003	GRMZM5G803812	Clone 1357151 mRNA sequence
ss196464985	PZE-105087408	5	112930947	1.76E-06	0.0012		
ss196418787	PUT-163a-16926285-1144	5	190211048	1.81E-05	0.0094	GRMZM2G004847	Grx_C3-glutaredoxin subgroup I
ss196523428	SYN38258	5	190748444	3.50E-08	0.0003	GRMZM2G171406	Putative uncharacterized protein
ss196467969	PZE-105134644	5	195303760	9.01E-07	0.0011	GRMZM2G032130	Putative uncharacterized protein
ss196470103	PZE-106015808	6	37859073	3.53E-08	0.0003		
ss196470162	PZE-106016518	6	39079387	1.05E-06	0.0011	GRMZM2G012964	Putative uncharacterized protein
ss196470391	PZE-106018502	6	44383530	1.20E-06	0.0011	GRMZM2G087570	Putative uncharacterized protein
ss196470900	PZE-106022774	6	55259192	1.38E-05	0.0073	GRMZM2G122261	Putative uncharacterized protein
ss196530306	SYNGENTA4261	6	58446196	1.14E-05	0.0061	GRMZM2G473788	Putative uncharacterized protein
ss196510095	SYN21708	6	133283261	1.52E-06	0.0011	GRMZM2G118587	Putative uncharacterized protein
ss196523284	SYN3810	7	5612721	1.51E-06	0.0011	GRMZM2G011314	Putative uncharacterized protein
ss196519912	SYN3390	7	164694172	1.12E-06	0.0011	GRMZM2G384528	Putative uncharacterized protein
ss196500671	SYN10192	8	25012274	1.51E-06	0.0011	GRMZM2G112686	
ss196482818	PZE-108030550	8	34486407	1.05E-06	0.0011		Esterase
ss196485395	PZE-108055598	8	100025485	9.23E-06	0.0050		
ss196485514	PZE-108057605	8	102710937	1.05E-06	0.0011	GRMZM2G164814	Amino acid carrier
ss196486847	PZE-108077747	8	133312993	1.43E-06	0.0011		
ss196486883	PZE-108078396	8	134073631	7.90E-07	0.0011	GRMZM2G047412	Putative uncharacterized protein
ss196488596	PZE-109000162	9	564361	2.22E-06	0.0015	GRMZM2G341658	Putative uncharacterized protein
ss196417097	sh1.1	9	11500578	1.10E-06	0.0011	GRMZM2G089713	Sucrose synthase 1 (EC 2.4.1.13)(Sucrose-UDP glucosyltransferase 1)(Shrunken-1)
ss196501966	SYN11758	9	14535446	1.51E-06	0.0011	GRMZM2G300408	Transposon protein
ss196417619	PZB02176.1	9	115594115	1.29E-06	0.0011		
ss196507634	SYN18761	10	109342928	1.23E-06	0.0011	GRMZM2G001805	Putative uncharacterized protein
ss196507629	SYN18759	10	109343086	2.36E-06	0.0015	GRMZM2G001805	Putative uncharacterized protein
ss196499043	PZE-110057895	10	111309556	1.05E-06	0.0011	GRMZM2G149590	Putative uncharacterized protein
ss196499169	PZE-110060367	10	114600883	1.48E-06	0.0011		
ss196499171	PZE-110060368	10	114621731	3.26E-06	0.0020		
ss196417470	PZA03607.3	10	142189571	6.76E-06	0.0038	GRMZM2G025592	DNA (cytosine-5)-methyltransferase 1 (EC 2.1.1.37)(Chromomethylase 1)(Zea methyltransferase2)(Zmet2)(DNA cytosine methyltransferase MET2a)
