# Supplementary material for: Spatial Structure and Climatic Adaptation in African Maize Revealed by Surveying SNP Diversity in Relation to Global Breeding and Landrace Panels
Source: PLoS One. 2012 Oct 16;7(10):e47832. doi: 10.1371/journal.pone.0047832 (PMC3472975; doi:10.1371/journal.pone.0047832)
Supplement: Figure S3 — Unrooted accession based NJ tree for the African panel based on 26,900 PZE-prefix SNPs. (PDF) [file pone.0047832.s003.pdf]

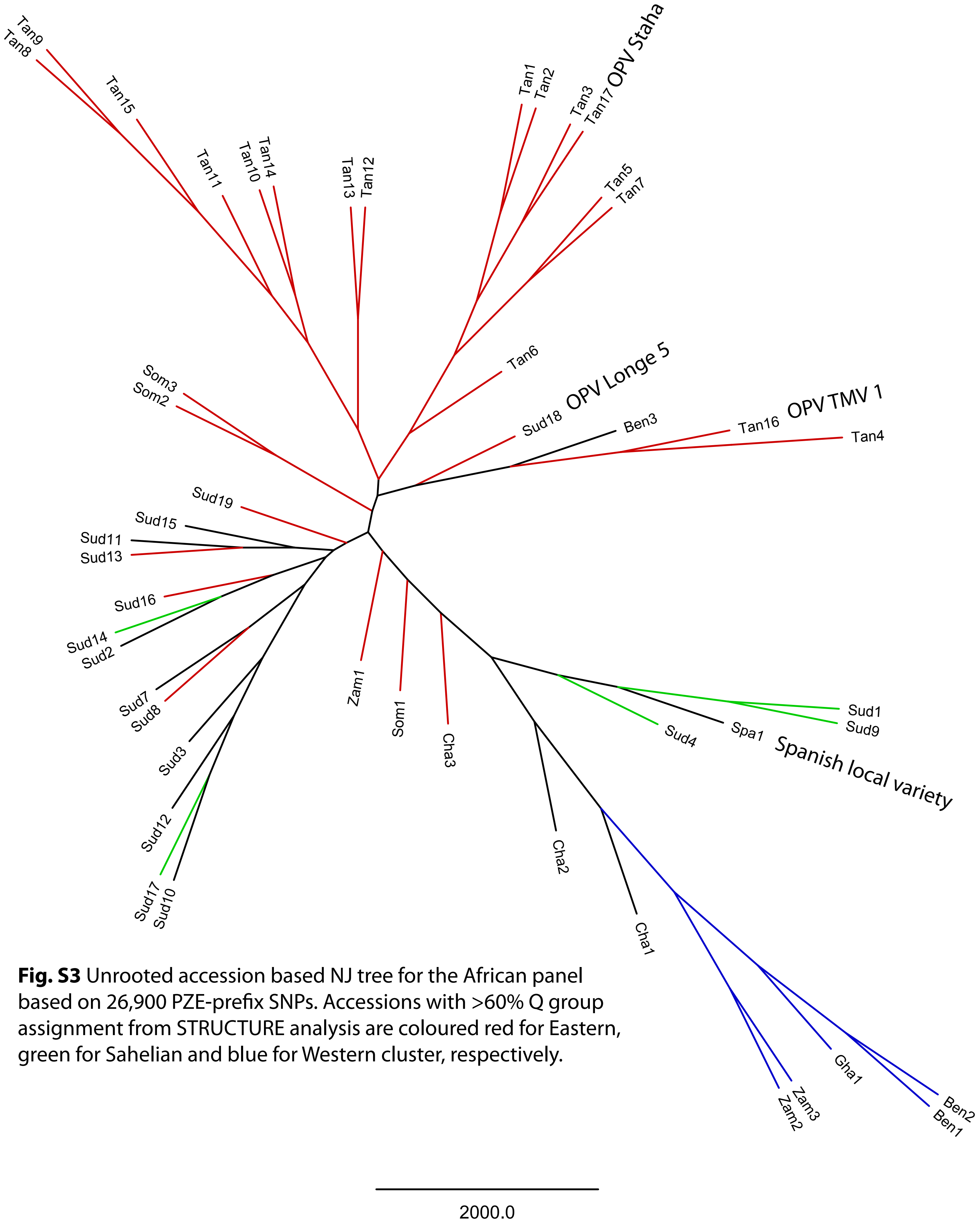

Fig. S3 Unrooted accession based NJ tree for the African panel based on 26,900 PZE-prefix SNPs. Accessions with >60% Q group assignment from STRUCTURE analysis are coloured red for Eastern, green for Sahelian and blue for Western cluster, respectively.
